# Relationship between residual cholesterol and cognitive performance: a study based on NHANES

**DOI:** 10.3389/fnut.2024.1458970

**Published:** 2024-09-11

**Authors:** Kepeng Liu, Haishou Fu, Yong Chen, Binfei Li, Huaqing Huang, Xiaozu Liao

**Affiliations:** ^1^Department of Anesthesiology, Zhongshan City People's Hospital, Zhongshan, Guangdong, China; ^2^Department of Clinical Laboratory, Fujian Provincial Hospital, Fuzhou University Affiliated Provincial Hospital, Fuzhou, Fujian, China; ^3^Department of Pain Medicine, Clinical Oncology School of Fujian Medical University, Fujian Cancer Hospital, Fuzhou, China

**Keywords:** remnant cholesterol, cognition performance, sleep, Z-scores, NHANES

## Abstract

**Background and aims:**

Age-related cognitive impairment impacts a significant portion of the elderly population. Remnant cholesterol (RC) has attracted increased attention in relation to cardiovascular disease, diabetes, hypertension, and fatty liver disease. Nevertheless, its role in cognitive function is still enigmatic, prompting our exploration into the potential associations between them.

**Methods:**

A total of 1,331 participants from the NHANES (2011–2014) database, all aged over 60, were included in this investigation. Cognitive function was assessed using four widely applied tests, including the Consortium to Establish a Registry for Alzheimer’s Disease Word Learning (CERAD-WL), CERAD Delayed Recall (CERAD-DR), Animal Fluency Test (AFT), as well as Digit Symbol Substitution test (DSST). Z-score is calculated by scores from the above four tests. The association between RC, total cholesterol (TC) to RC and cognitive performance was assessed by logistic regression analyses. In addition, restricted cubic spline (RCS) regression was performed to assess non-linearity between RC and cognitive function. Subgroup analysis was performed to evaluate the robustness of the results in populations with relevant covariate variables.

**Results:**

Those with Z-scores below the 25% quartile are defined as having cognitive impairment, totaling 498 individuals. Observationally, higher RC levels and a lower TC/RC were associated with an increased risk of cognitive impairment. After adjusting for confounding factors, the impact of RC levels on cognitive performance quartiles was consistent across various subgroups, except in individuals with trouble sleeping, no/unknown alcohol use, and no hypertension. Americans with high RC levels and trouble sleeping are more likely to develop cognitive impairment, with an odds ratio of 2.33 (95% CI: 1.18–4.59).

**Conclusion:**

This study suggests that higher RC levels and lower levels of TC/RC are associated with an increased likelihood of cognitive impairment, suggesting that RC can serve as a novel and convenient indicator for predicting the risk of cognitive impairment in the US population.

## 1 Introduction

Cognition is defined as the operation of the mind with all facets of perceiving, thinking, and remembering, commonly assessed by a diverse range of neuropsychological tests (1, 2). In the aging process, although there is often a decrease in the performance of some cognitive functions, this does not necessarily imply a diagnosis of mild cognitive impairment (MCI) or dementia; it is a possibility rather than a certainty. Cognitive impairment can occur in some individuals as they age, potentially leading to MCI or even dementia (3). MCI impacts approximately 16 to 20% of older adults (4). Furthermore, over a five-year span, more than one-third of individuals with MCI progress to develop dementia (5). Cognitive impairment and dementia affect millions of people globally, placing substantial financial burdens on families and healthcare systems (6). While lifestyle modifications have been posited as potentially effective, no pharmacologic treatments have demonstrated efficacy in slowing the progression from MCI to dementia (7, 8). Therefore, discerning risk factors for cognitive impairment could contribute to slowing or preventing the onset of dementia.

Lipids include triacylglycerols (TGs), low-density lipoprotein (LDL), high-density lipoprotein (HDL), and total cholesterol (TC) (9). Extensive research established that uprise blood lipids play a pivotal role in atherosclerotic cardiovascular diseases (10), metabolic disorders (11, 12), immune regulation (11), and cancer (13). Recent investigations (14, 15) have broadened their scope to scrutinize the influence of these lipids on cognitive function and sleep. Preceding studies have accentuated a genetically dependent and nonlinear relationship between blood lipoproteins and cognitive function (16, 17). Remnant cholesterol (RC), has garnered heightened attention and reveals robust associations with prevalent diseases, such as cardiovascular disease (18–21), diabetes (22), hypertension (23), and fatty liver disease (24). Elevated levels of RC are closely related to TC metabolism disorders and insufficient hepatic clearance of residual lipoproteins. Meanwhile, APOE variants are closely associated with the risk of Alzheimer’s disease (AD) and are involved in the regulation of lipid metabolism in the liver (25). Therefore, RC may affect cognitive function.

There exists a scarcity of studies leveraging the National Health and Nutrition Examination Survey (NHANES) database to delve into the potential relationship between RC and cognitive function. The aim of this study was to elucidate and clarify the correlation between RC levels and the presence of cognitive function among NHANES participants. We hypothesized that individuals diagnosed with cognitive impairment would exhibit significantly elevated RC levels.

## 2 Materials and methods

### 2.1 Study population

The data were retrieved from the National Health and Nutrition Examination Survey (NHANES) database,^1^ which employs a complex survey design and population-specific sample weights to assess the health or nutritional status of the non-institutionalized population in the United States. The assessment methodology includes a series of sampled household interviews and standardized physical examinations conducted at Mobile Examination Centers (MECs). We retrieved health examination results, laboratory samples, and interviewed personal information from cognitive function surveys conducted among adults aged 60 and over from 2011 to 2014. All participants signed informed consent forms, and the study was approved by the Research Ethics Review Board of the National Center for Health Statistics.

**Figure 1:**
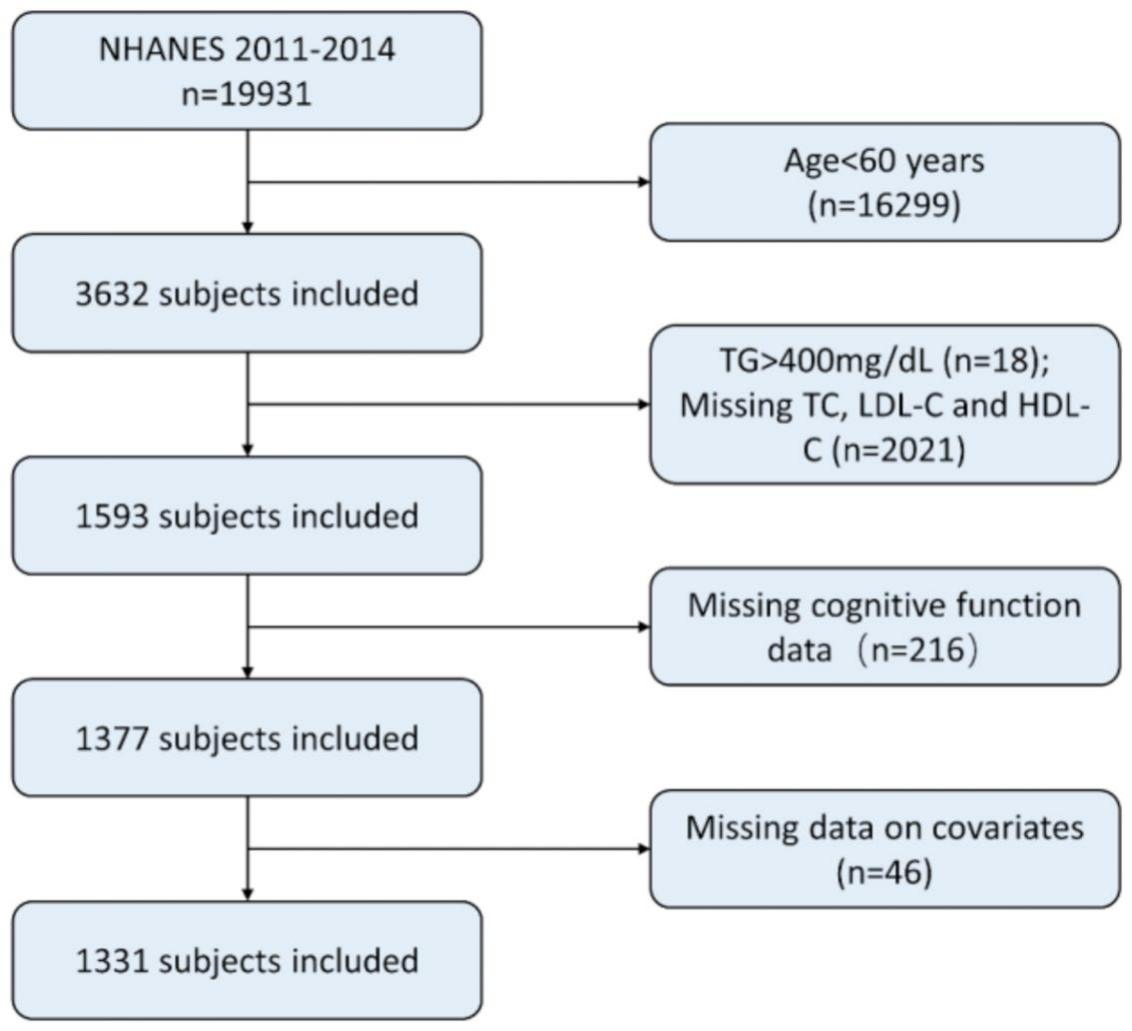
Patient selection. NHANES, National Health and Nutrition Examination Survey; TG, Triacylglycerol; LDL-C, Low-density lipoprotein cholesterol; HDL-C, High-density lipoprotein cholesterol.

We excluded (1) adults aged under 60 years old. (2) individuals with TG higher than 400 mg/dL. (3) individuals with missing laboratory data on HDL-C, LDL-C, or TC; (4) individuals without cognitive data; (5) individuals with missing data on other co-variables, such as BMI, smoking status, diabetes, and hypertension. A total of 1,331 individuals were ultimately included in this study. The process of recruiting is shown in Figure 1.

### 2.2 Assessment of cognitive performance

Cognitive function was evaluated by four widely applied tests, which included the Consortium to Establish a Registry for Alzheimer’s Disease Word Learning (CERAD-WL), CERAD Delayed Recall (CERAD-DR), Animal Fluency Test (AFT), as well as Digit Symbol Substitution test (DSST) (26). CERAD, designed to identify Alzheimer’s disease, is a highly reliable and valid cognitive function evaluation tool (27). The CERAD-WL involves three consecutive learning trials and an additional delayed recall trial. Participants read 10 irrelevant words in the first three trials and immediately recall as many as possible in a disrupted order each trial. Each correct word scores 1, and the total score is 30 (26). The AFT assesses verbal fluency, where participants are asked to name as many animals as possible in 1 min. A score of less than 14 indicates a decrease in cognitive function (28). The DSST test is a global measure of brain health, evaluating processing speed, visual scanning, sustained attention, and short-term memory (29). In the NHANES study, the version of The Digit Symbol Substitution Test (DSST) used is from the Wechsler Adult Intelligence Scale (WAIS III), with a time limit of 120 s. Participants have 2 min to match symbols with numbers in 133 boxes. Each correct match scores one point. A score greater than 33 indicates healthy cognitive function.

Z-score is calculated by scores from the above four tests for a more reliable and comprehensive evaluation of cognitive function. Here is the formula *Z* = (x–m)/σ(x for the raw score, m for overall mean, and σ for the overall standard deviation) (30). Recognizing the substantial impact of age on cognitive function, age-stratified quartiles of Z-scores (≥80 years, ≥70 to 80 years, and ≥ 60 to 70 years,) were used to assess cognitive performance. Subsequently, participants were classified into four cognitive function tiers (1 to 4) based on weighted quartiles derived from Z-scores within these age groups. The bottom quartile indicated cognitive impairment, with ascending quartiles corresponding to normal cognitive function.

### 2.3 Sleep problems

Sleep duration (14, 31) was self-reported by the question, “How much sleep do you get (hours)?.” Trouble sleeping was evaluated by the answer to “Have you ever told a doctor or other health professional that you have trouble sleeping?.” Sleep disorder was assessed by the response to “Have you ever been told by a doctor or other health professional that you have a sleep disorder?.” The responses to this question have been categorized into two distinct options: ‘yes’ or ‘no’.

### 2.4 Lipids assessment

In the NHANES laboratory, serum or plasma TC, HDL-C, and LDL-C were measured in participants who had fasted for more than 8.5 h but less than 24 h. TC and TGs were measured using an enzymatic assay method. HDL-C was measured using the heparin-manganese precipitation method or a direct immunoassay technique. Due to technological limitations, the precise numerical value of TG exceeding 400 mg/dL cannot be accurately displayed. When the TG value was 400 mg/dL or less, LDL-C was calculated by the following formula [LDL-C] = [TC]—[HDL-C]—[TG/5] according to the following Friedwald formula (32, 33). RC and TC/RC were the exposure variables. RC = TC—(LDL-C + HDL-C) (17). The TC/RC stands for TC divided by RC.

### 2.5 Co-variables

We collected potential confounding factors from standardized questionnaires. The data contained gender, age, race, education level, marital status, body mass index (BMI), alcohol use, smoking status, diabetes, and hypertension. Self-reported race was categorized into five groups: non-Hispanic White, non-Hispanic Black, Mexican American, other/multiracial race, and other/Hispanic race. Educational level was stratified into three categories: less than high school, high school or equivalent, and college or above. Marital status was divided into two groups: married and unmarried. BMI was categorized into four groups: underweight (<18.5), normal (18.5 to <25), overweight (25 to <30), and obese (30 or greater). Alcohol use was classified into two groups: no/unknown and yes. Smoking status was defined as current smoker, former smoker, and never smoker. Sleep duration was categorized as short (less than 7 h per night), normal (7–9 h per night), or long (greater than 9 h per night). Participants with diabetes were identified if they had any of the following conditions: diagnosed by a doctor or health professional, HbA1c ≥ 6.5%, fasting blood glucose ≥7.0 mmoL/L, random blood glucose ≥11.1 mmoL/L, 2-h OGTT blood glucose ≥11.1 mmoL/L, or use of diabetes medication or insulin. Hypertension was defined based on any of the following: diagnosed by a doctor or health professional, average systolic blood pressure ≥ 140 mmHg, or average diastolic blood pressure ≥ 90 mmHg.

### 2.6 Statistical analysis

The R software version 4.3.1 was utilized for the analysis of NHANES data, taking into account the intricate sampling design and corresponding sampling weights. The sampling weights for the combined four-year cycles were derived by dividing the individual sampling weights by 2. The data were presented as the mean ± standard deviation for continuous variables, median with interquartile range for skewed variables, and sample counts with weighted percentages for categorical variables. To fit the regression models, TC/RC and continuous RC values were subjected to natural log-transformation. Categorical RC and TC/RC were divided into four subgroups using quartiles as cutoff points.

To evaluate the association between RC, TC/RC, and cognitive performance, we conducted weighted logistic regression analyses. Sequential models were adjusted for potential risk factors: Model 1 without covariate adjustments, Model 2 adjusted for age, gender, and race, Model 3 further including education, marital status, and BMI in addition to the variables in Model 2, and Model 4 incorporating all co-variables such as alcohol use, smoking status, diabetes, and hypertension, along with the adjustments made in Model 3.

In order to assess the robustness of our analytical results, we conducted sensitivity analyses using logistic regression based on unweighted raw data. Additionally, to assess nonlinearity and confirm the dose–response relationship between residual cholesterol levels and cognitive function, a restricted cubic spline (RCS) regression analysis was performed, accounting for all variables in Model 4. Subgroup analysis was conducted to validate the robustness of the results in populations with specific covariate variables. A *p*-value of less than 0.05 was deemed statistically significant.

## 3 Results

### 3.1 Characteristics of the study population

Table 1 outlines the sociodemographic and baseline characteristics. 1,331 individuals aged 60 years or older were included. The median age was 68, with females constituting 56% of the cohort. Cognitive impairment was identified in 498 subjects. Populations with different RC levels had similar prevalence rates in terms of age, sex, education level, marital status, trouble sleeping, sleep duration, smoking status, and alcohol-related conditions. However, there were significant differences in the prevalence of race, BMI, sleep disorder, diabetes, hypertension, Z-score, and cognitive function. The prevalence rates of cognitive impairment across the quartiles of RC levels were 18, 23, 33, and 27%, respectively.

**Table 1:** Baseline characteristics.

Characteristic	Overall, *N* = 1,331 (100%)^a^	Q1, *N* = 364 (25%)	Q2, *N* = 329 (25%)	Q3, *N* = 307 (25%)	Q4, *N* = 331 (25%)	*p* Value^b^
**Age (years)**	68 (63, 74)	68 (63, 74)	68 (64, 76)	69 (64, 75)	67 (64, 74)	0.13
**Sex**						0.5
Female	685 (56%)	188 (56%)	167 (53%)	153 (53%)	177 (59%)	
Male	646 (44%)	176 (44%)	162 (47%)	154 (47%)	154 (41%)	
**Age**						0.057
< 70 years	709 (58%)	203 (61%)	172 (56%)	151 (53%)	183 (60%)	
70–80 years	401 (28%)	93 (22%)	97 (28%)	108 (34%)	103 (30%)	
≥80 years	221 (14%)	68 (17%)	60 (15%)	48 (13%)	45 (11%)	
**Race, *n* (%)**						**0.001**
Non-Hispanic White	671 (80%)	178 (79%)	160 (79%)	146 (77%)	187 (83%)	
Non-Hispanic Black	269 (8.3%)	113 (13%)	68 (9.2%)	53 (7.8%)	35 (3.6%)	
Other Hispanic	141 (3.5%)	27 (2.6%)	30 (3.0%)	47 (5.5%)	37 (3.5%)	
Other/multiracial	131 (5.0%)	31 (4.1%)	36 (4.4%)	31 (6.0%)	33 (5.8%)	
Mexican American	119 (3.5%)	15 (1.5%)	35 (4.4%)	30 (4.2%)	39 (4.3%)	
**Education level**						0.14
Less than high school	340 (16%)	77 (12%)	80 (16%)	92 (22%)	91 (16%)	
High school or equivalent	314 (22%)	81 (19%)	77 (22%)	64 (21%)	92 (28%)	
College or above	677 (61%)	206 (69%)	172 (62%)	151 (57%)	148 (56%)	
**Marital status, *n* (%)**						0.4
Married	786 (65%)	213 (68%)	204 (69%)	176 (61%)	193 (63%)	
Unmarried	545 (35%)	151 (32%)	125 (31%)	131 (39%)	138 (37%)	
**BMI (kg/m** ^**2**^ **)**						**<0.001**
Underweight (<18.5)	17 (1.2%)	7 (2.4%)	5 (0.6%)	3 (0.6%)	2 (1.0%)	
Normal (18.5 to < 25)	353 (26%)	135 (42%)	90 (26%)	78 (23%)	50 (13%)	
Overweight (25 to < 30)	455 (35%)	117 (34%)	133 (43%)	104 (36%)	101 (28%)	
Obese (30 or greater)	506 (38%)	105 (22%)	101 (30%)	122 (41%)	178 (59%)	
**Trouble sleeping**						0.5
Non-trouble sleeping	939 (68%)	269 (72%)	231 (69%)	214 (68%)	225 (66%)	
Trouble sleeping	392 (32%)	95 (27%)	98 (30%)	93 (31%)	106 (33%)	
**Sleep disorder, *n* (%)**						**0.005**
Sleep disorder	165 (13%)	37 (10%)	35 (8.6%)	35 (13%)	58 (20%)	
Non-sleep disorder	1,166 (87%)	327 (90%)	294 (91%)	272 (87%)	273 (80%)	
**Sleep duration**						0.6
< 7 h	435 (27%)	111 (26%)	117 (28%)	100 (27%)	107 (27%)	
7–9 h	772 (65%)	223 (66%)	187 (67%)	177 (64%)	185 (62%)	
≥9 h	124 (8.4%)	30 (8.3%)	25 (5.4%)	30 (9.2%)	39 (11%)	
**Smoke status, *n* (%)**						0.3
Current smoker	159 (11%)	38 (8.2%)	35 (7.9%)	43 (14%)	43 (13%)	
Former smoker	507 (40%)	141 (40%)	114 (38%)	126 (43%)	126 (40%)	
Never smoker	665 (49%)	185 (52%)	180 (54%)	138 (43%)	162 (47%)	
**Alcohol, *n* (%)**						0.3
No/unknown	213 (14%)	44 (12%)	70 (17%)	47 (14%)	52 (11%)	
Yes	1,118 (86%)	320 (88%)	259 (83%)	260 (86%)	279 (89%)	
**Diabetes, *n* (%)**						**0.011**
No	980 (77%)	290 (87%)	246 (76%)	222 (77%)	222 (69%)	
Yes	351 (23%)	74 (13%)	83 (24%)	85 (23%)	109 (31%)	
**Hypertension, *n* (%)**						**<0.001**
No	499 (41%)	149 (51%)	147 (49%)	109 (35%)	94 (28%)	
Yes	832 (59%)	215 (49%)	182 (51%)	198 (65%)	237 (72%)	
**WL**	20 (17, 23)	22 (18, 24)	21 (18, 23)	19 (16, 22)	20 (17, 23)	**0.003**
**DR**	7 (5, 8)	7 (5, 8)	6 (5, 8)	6 (5, 8)	6 (5, 8)	0.078
**AF**	18 (14, 21)	18 (14, 22)	18 (14, 21)	17 (14, 21)	17 (14, 21)	0.7
**DSST**	53 (41, 64)	56 (41, 67)	50 (40, 63)	48 (36, 59)	55 (44, 63)	**0.004**
**Z-score**	0.39 (−0.32, 1.00)	0.65 (−0.19, 1.17)	0.31 (−0.40, 0.97)	0.23 (−0.48, 0.77)	0.42 (−0.21, 0.98)	**0.013**
**Cognitive function**						0.012
Normal	833 (75%)	250 (82%)	200 (77%)	174 (67%)	209 (73%)	
Decline	498 (25%)	114 (18%)	129 (23%)	133 (33%)	122 (27%)	

BMI, Body mass index; CERAD-WL, Consortium to Establish a Registry for Alzheimer’s Disease Word Learning; CERAD-DR, CERAD Delayed Recall; AFT, Animal Fluency Test; DSST, Digit Symbol Substitution test; TC, Total cholesterol; TG, Triacylglycerol; LDL-C, Low-density lipoprotein cholesterol; HDL-C, High-density lipoprotein cholesterol; RC, Remnant cholesterol.

a Median (IQR) for continuous; *n* (%) for categorical.

b Wilcoxon rank-sum test for complex survey samples; chi-squared test with Rao and Scott’s second-order correction.

Significant differences were observed in age, race, education level, marital status, smoking status, diabetes, sleep duration, HDL-C, RC, LDL-C, TC, and TC/RC between the normal and cognitive impairment groups (Supplementary Table S1). Participants with cognitive impairment exhibited lower levels of TC, LDL-C, HDL-C, and TC/RC (all *p* < 0.05) and higher levels of RC (*p* = 0.048). In the cognitive normal group, individuals with prolonged sleep (≥ 9 h) accounted for 13%, surpassing the 6.9% in the cognitive impairment group.

### 3.2 The association among RC, TC/RC and cognitive performance

**Figure 2:**
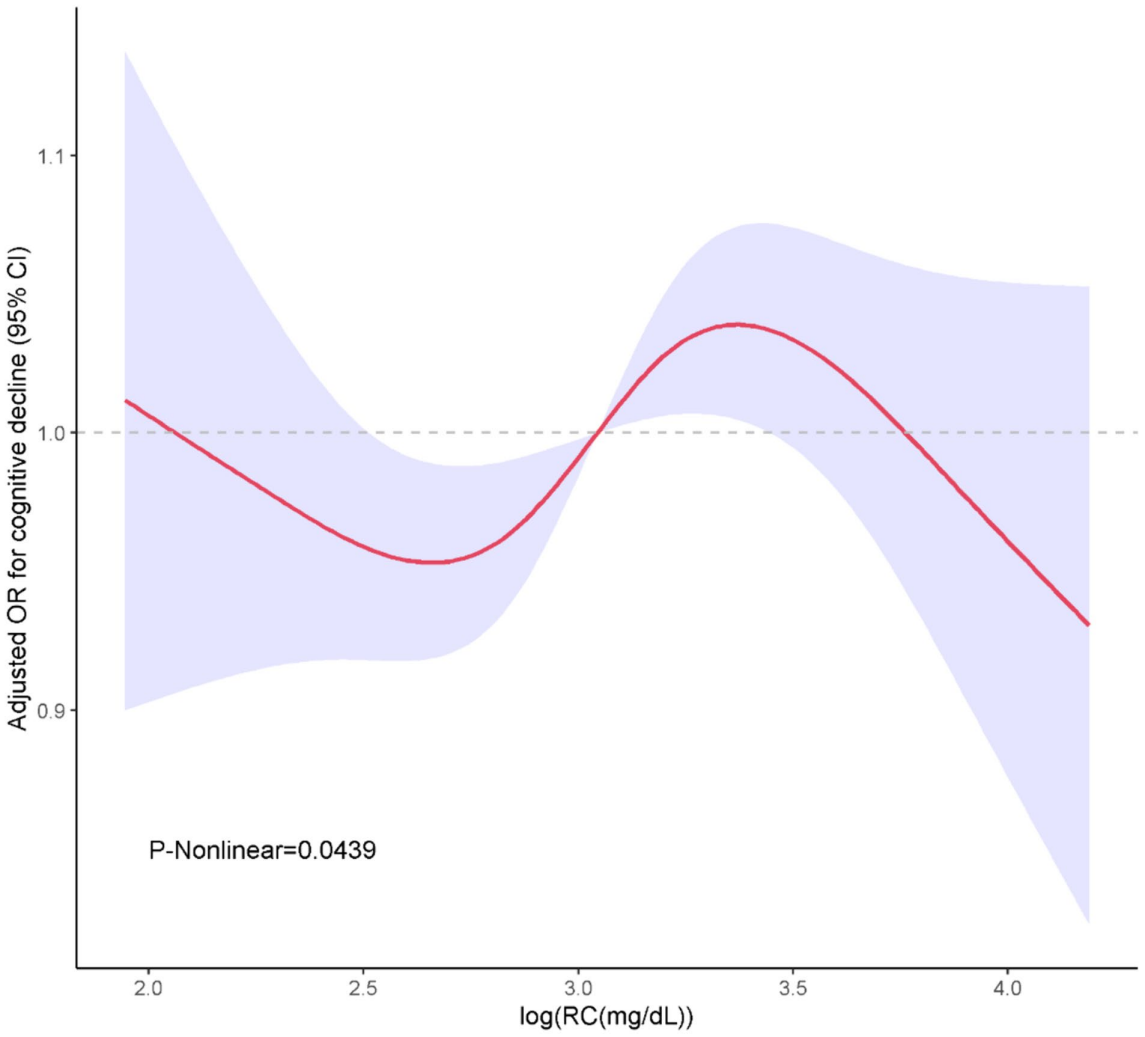
Restricted cubic spline curves for association of RC with the cognitive performance.

In Table 2, we delineated the relationships between RC, TC/RC and cognitive performance. Participants were stratified based on the interquartile range of RC levels. The lowest quartile (Q1) was the referential baseline. A statistically significant positive association was identified between RC and cognitive performance at the third quartile (Q3) in all analytical models examined. Specifically, for Model 1, the odds ratio (OR) with 95% confidence interval (CI) was calculated as 2.2 (1.43, 3.37), with a *p*-value of less than 0.001. Similarly, Model 2 exhibited an OR of 2.31 (1.45, 3.69), *p* = 0.001; Model 3 showed an OR of 2.21 (1.45, 3.37), *p* = 0.001; and Model 4 demonstrated an OR of 2.11 (1.37, 3.25), *p* = 0.003. Furthermore, a positive association between TC/RC and cognitive performance was also identified at the Q3 level in Model 4, with an OR of 0.56 (0.33, 0.94) and a p-value of 0.032. Additionally, a non-linear correlation between RC and cognitive performance was observed within restricted cubic spline (RCS) regression model limitations (P-nonlinear = 0.0439) as depicted in Figure 2.

**Table 2:** Association of RC (log-transformed) and TC/RC (log-transformed) with cognitive performance.

Characteristic		Model 1		Model 2		Model 3		Model 4	
	Cognitive dysfunction (*n*%)	OR^1^ (95% CI^1^)	*P* value	OR^1^ (95% CI^1^)	*P* value	OR^1^ (95% CI^1^)	*P* value	OR^1^ (95% CI^1^)	*P* value
**RC**									
Q1(1.61–2.71)	114 (20%)	Ref		Ref		Ref		Ref	
Q2 (2.71–3.04)	129 (22%)	1.35 (0.85–2.14)	0.194	1.38 (0.84–2.27)	0.189	1.39 (0.83–2.33)	0.197	1.35 (0.78–2.32)	0.3
Q3 (3.04–3.37)	133 (29%)	2.20 (1.43–3.37)	<0.001	2.31 (1.45–3.69)	0.001	2.21 (1.45–3.37)	0.001	2.10 (1.36–3.25)	0.003
Q4 (3.37–4.34)	122 (29%)	1.62 (0.97–2.69)	0.063	1.94 (1.10–3.40)	0.023	1.85 (1.01–3.38)	0.046	1.69 (0.91–3.15)	0.088
p for trend		0.001		0.001		<0.001		<0.001	
**TC/RC**									
Q1 (0.34–1.84)	133 (32%)	Ref		Ref		Ref		Ref	
Q2 (1.84–2.17)	146 (30%)	1.16 (0.70–1.93)	0.557	1.09 (0.63–1.91)	0.739	1.02 (0.53–1.95)	0.950	1.08 (0.55–2.11)	0.809
Q3 (2.17–2.52)	115 (20%)	0.71 (0.49–1.02)	0.062	0.58 (0.39–0.87)	0.011	0.51 (0.30–0.86)	0.014	0.56 (0.33–0.95)	0.035
Q4 (2.52–3.77)	104 (19%)	0.52 (0.29–0.94)	0.031	0.48 (0.25–0.92)	0.029	0.49 (0.23–1.04)	0.06	0.55 (0.26–1.19)	0.116
p for trend		<0.001		<0.001		<0.001		<0.001	

Model 1 univariable analysis. Model 2 adjusted for age, gender, and race. Model 3 further adjusted for education, marital status and BMI status plus model 2. Model 4 included adjustments for alcohol use, smoking status, trouble sleeping, diabetes, and hypertension plus model 3. OR, Odds ratio; CI, Confidence interval; RC, Remnant cholesterol; TC, Total cholesterol.

### 3.3 Sensitivity analysis

Similarly, sensitivity analyses using unweighted logistic analyses showed that individuals with the highest quartile of the RC had a lower cognitive decline than those with the lowest quartile of the RC, according to the different models, Model 1 (OR = 1.28, 95% CI: 0.93–1.75, *p* < 0.12), Model 2 (OR = 1.52, 95% CI:1.08–2.14, *p* < 0.017), and Model 3 (OR = 1.47, 95% CI: 1.01–2.14, *p* < 0.046) (Table 3). The RC and cognitive performance appear to consistently correlate negatively, according to these findings.

**Table 3:** Association of unweighted RC (log-transformed) and TC/RC (log-transformed) with cognitive performance.

Characteristic		Model 1		Model 2		Model 3		Model 4	
	Cognitive dysfunction (n%)	OR^1^ (95% CI^1^)	*P* value	OR^1^ (95% CI^1^)	*P* value	OR^1^ (95% CI^1^)	*P* value	OR^1^ (95% CI^1^)	*P* value
**RC**									
Q1(1.61–2.71)	114 (20%)	Ref		Ref		Ref		Ref	
Q2 (2.71–3.04)	129 (22%)	1.41 (1.03–1.94)	0.030	1.50 (1.08–2.10)	0.017	1.58 (1.10–2.26)	0.013	1.52 (1.06–2.19)	0.023
Q3 (3.04–3.37)	133 (29%)	1.68 (1.22–2.30)	0.001	1.75 (1.25–2.47)	0.001	1.76 (1.22–2.55)	0.003	1.70 (1.17–2.47)	0.005
Q4 (3.37–4.34)	122 (29%)	1.28 (0.93–1.75)	0.12	1.52 (1.08–2.14)	0.017	1.47 (1.01–2.14)	0.046	1.38 (0.95–2.03)	0.095
p for trend		0.012		0.008		0.015		0.03	
**TC/RC**									
Q1 (0.34–1.84)	133 (32%)	Ref		Ref		Ref		Ref	
Q2 (1.84–2.17)	146 (30%)	1.17 (0.86–1.59)	0.3	1.09 (0.79–1.52)	0.6	1.05 (0.74–1.50)	0.778	1.08 (0.75–1.54)	0.679
Q3 (2.17–2.52)	115 (20%)	0.8 (0.58–1.09)	0.2	0.66 (0.47–0.93)	0.018	0.64 (0.44–0.93)	0.020	0.69 (0.47–0.99)	0.049
Q4 (2.52–3.77)	104 (19%)	0.69 (0.50–0.94)	0.021	0.63 (0.44–0.89)	0.009	0.63 (0.43–0.93)	0.020	0.68 (0.45–1.00)	0.053
p for trend		0.005		0.001		0.007		<0.001	

Model 1 univariable analysis. Model 2 adjusted for age, gender, and race. Model 3 further adjusted for education, marital status and BMI status plus model 2. Model 4 included adjustments for alcohol use, smoking status, trouble sleeping, diabetes, and hypertension plus model 3. OR, Odds ratio; CI, Confidence interval; RC, Remnant cholesterol; TC, Total cholesterol.

### 3.4 Subgroup analysis

**Figure 3:**
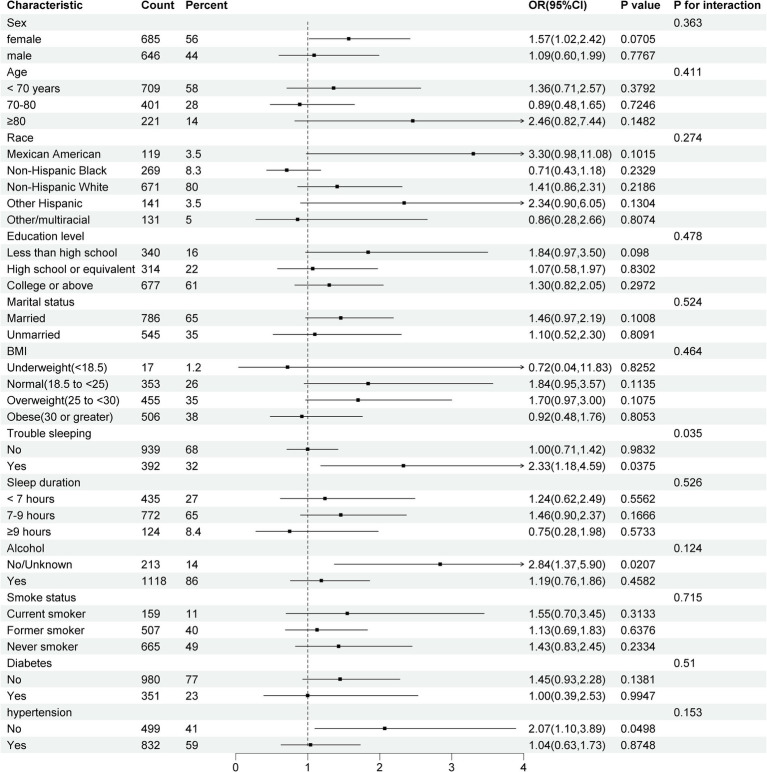
Forest plot for subgroup analyses of the association between RC and cognitive performance. Model adjusted for age, gender, race, education, marital status, BMI status, alcohol use, smoking status, diabetes, and hypertension. CI, Confidence interval; BMI, Body mass index.

After adjusting for the full model in these subgroups, a significant interaction emerged between RC levels and cognitive impairment specifically among individuals experiencing trouble sleeping (*p* = 0.035). Americans with higher RC levels who also struggle with sleep difficulties may face an increased risk of cognitive impairment, with an odds ratio of 2.33 (95% CI, 1.18–4.59). Conversely, no such interactions were identified in other subgroups (*p* > 0.05) in Figure 3. After adjusting for confounding factors, the impact of RC levels on cognitive performance quartiles was consistent across various subgroups, including gender, age, race, education level, marital status, BMI, sleep duration, alcohol use, smoking, diabetes, and hypertension (*p* ≥ 0.05). Exceptions were noted in individuals with trouble sleeping, no/unknown alcohol use, and no hypertension, where the *p*-values <0.05.

## 4 Discussion

To our knowledge, this study is the first to investigate the association between plasma RC levels and TC/RC with cognitive performance in a nationally representative U.S. cohort. Our findings indicated that higher RC levels and lower levels of TC/RC were associated with positive likelihood of cognitive impairment. Even after adjusting for race, gender, age, education, marital status, BMI, alcohol use, smoking status, diabetes, and hypertension, elevated RC levels and a low TC/RC ratio were linked to greater cognitive impairment. The effect sizes of RC levels as well as the TC/RC ratio on cognitive impairment were consistent across almost all subgroup analyses except trouble sleeping. Higher RC levels may increase the risk of cognitive impairment among Americans who experience trouble sleeping.

There is a scarcity of previous research examining the relationship between RC and cognitive function in sizeable and diverse representative samples. Xie et al. (31) reported an association between higher RC levels and lower TC to RC ratios with increased risk of verbal learning and memory dysfunction. Analysis of 5,358 postmenopausal women from the Women’s Health Initiative Memory Study showed higher RC in individuals with Apolipoprotein E4 (apoE4) mutation, the most prevalent genetic risk factor of AD, was linked to increased dementia and cognitive impairment risk compared with lower RC group (34). These studies primarily utilized CERAD total scores for cognitive assessment. Our study adopted a more comprehensive methodology, utilizing a Z-score, which was integrated by the popularly and widely used four cognitive tests, potentially offering a more reliable and comprehensive evaluation of cognitive function. Moreover, prior research has demonstrated that trouble sleeping is inclined to facilitate cognitive impairment (35–37). We have incorporated sleep as a crucial factor to investigate the effect of RC on cognitive performance. Our findings reveal that RC has a noteworthy influence on cognitive function among individuals struggling with sleep difficulties.

The effects of RC metabolism on cognitive function might be attributed to its damage to brain cells. Cholesterol is crucial for cellular membranes, signaling pathways, and as a precursor for oxysterols and hormones (32, 33). Additionally, RC, smaller than 70 nanometers, could penetrate arterial walls, contributing to atherosclerosis in cerebral arteries and arterioles, potentially impairing cognitive function by reducing cerebral blood flow (38, 39). However, more animal experiments are needed to confirm our speculation.

The onset age of dyslipidemia might be related to the development of cognitive impairment. A recent meta-analysis, synthesizing data from over 23,000 patients across 17 studies, revealed that the highest risk for developing AD in association with hypercholesterolemia occurs during midlife and the early stages of aging (40), while no significant association is observed if the hypercholesterolemia occurs in later life (41). To fully understand lipids’ impact on late-life brain health, extensive follow-ups of cohorts with cognitive impairment are needed.

### 4.1 Strengths and limitation

The study presented several notable strengths, including a substantial sample size and the utilization of Z-scores as a metric for cognitive function evaluation. However, there are some limitations. Firstly, some older adults potentially experiencing cognitive impairment might have been excluded if they were unable to complete cognitive function assessments. Additionally, this research, grounded in the U.S. population and database, necessitates a broader inclusion of diverse regions and further clinical trials to validate the role of RC in cognitive function more conclusively. Since most of the data is self-reported, an information bias may have been induced. What’s more, given the cross-sectional design and lack of follow-up, it is not impossible that a reverse causality bias has been introduced.

## 5 Conclusion

In conclusion, higher levels of RC and lower levels of TC/RC are associated with an increased likelihood of cognitive impairment, suggesting that RC can serve as a novel and convenient indicator for predicting the risk of cognitive impairment in the US population. Furthermore, these findings provide insights into early intervention strategies for vulnerable populations who are at risk of developing cognitive impairment.

## Data Availability

The original contributions presented in the study are included in the article/Supplementary material, further inquiries can be directed to the corresponding authors.

## Footnotes

^1^ https://www.cdc.gov/nchs/nhanes/

## References

[1] Dorland. Dorland's Illustrated Medical Dictionary. Philadelphia: W.B. Saunders (1925).

[2] Montine T. J. Bukhari S. A., And White L. R. (2021). Cognitive impairment in older adults and therapeutic strategies. Pharmacol Rev 73, 152–162. doi: 10.1124/Pharmrev.120.000031, PMID: 33298513 PMC7736830

[3] Morley JE. An overview of cognitive impairment. Clin Geriatr Med. (2018) 34:505–13. doi: 10.1016/J.Cger.2018.06.003 30336985

[4] Roberts R., And Knopman D. S. (2013). Classification and epidemiology of mci. Clin Geriatr Med 29, 753–772. doi: 10.1016/J.Cger.2013.07.003, PMID: 24094295 PMC3821397

[5] Guo J Wang J Dove A Chen H Yuan C Bennett DA. Body mass index trajectories preceding incident mild cognitive impairment and dementia. JAMA Psychiatry. (2022) 79:1180–7. doi: 10.1001/Jamapsychiatry.2022.3446, PMID: 36287554 PMC9608028

[6] Pérez Palmer N. Trejo Ortega B., And Joshi P. (2022). Cognitive impairment in older adults: epidemiology, diagnosis, and treatment. Psychiatr Clin North Am 45, 639–661. doi: 10.1016/J.Psc.2022.07.010 36396270

[7] Blackman J. Swirski M. Clynes J. Harding S. Leng Y., And Coulthard E. (2021). Pharmacological and non-pharmacological interventions to enhance sleep in mild cognitive impairment and mild Alzheimer's disease: a systematic review. J Sleep Res 30,:E13229. doi: 10.1111/Jsr.13229, PMID: 33289311 PMC8365694

[8] Sanford AM. Mild cognitive impairment. Clin Geriatr Med. (2017) 33:325–37. doi: 10.1016/J.Cger.2017.02.005 28689566

[9] Pakiet A. Kobiela J. Stepnowski P. Sledzinski T., And Mika A. (2019). Changes in lipids composition and metabolism in colorectal Cancer: a review. Lipids Health Dis 18,:29. doi: 10.1186/S12944-019-0977-8, PMID: 30684960 PMC6347819

[10] Li Z Zhu G Chen G Luo M Liu X Chen Z. Distribution of lipid levels and prevalence of hyperlipidemia: data from the Nhanes 2007-2018. Lipids Health Dis. (2022) 21:111. doi: 10.1186/S12944-022-01721-Y, PMID: 36307819 PMC9615374

[11] Lim S. A. Su W. Chapman N. M., And Chi H. (2022). Lipid metabolism in T cell signaling and function. Nat Chem Biol 18, 470–481. doi: 10.1038/S41589-022-01017-3, PMID: 35484263 PMC11103273

[12] Sun Y Zhou S Guo H Zhang J Ma T Zheng Y. Protective effects of Sulforaphane on type 2 diabetes-induced cardiomyopathy via Ampk-mediated activation of lipid metabolic pathways and Nrf2 function. Metabolism. (2020) 102:154002. doi: 10.1016/J.Metabol.2019.154002, PMID: 31706979

[13] Bian X. Liu R. Meng Y. Xing D. Xu D., And Lu Z. (2021). Lipid metabolism and Cancer. J Exp Med 12,:784. doi: 10.1084/Jem.20201606, PMID: 33601415 PMC7754673

[14] Yap CX Henders AK Alvares GA Giles C Huynh K Nguyen A. Interactions between the Lipidome and genetic and environmental factors in autism. Nat Med. (2023) 29:936–49. doi: 10.1038/S41591-023-02271-1, PMID: 37076741 PMC10115648

[15] Zhao M. Tuo H. Wang S., And Zhao L. (2020). The effects of dietary nutrition on sleep and sleep disorders. Mediat Inflamm 2020,:3142874. doi: 10.1155/2020/3142874, PMID: 32684833 PMC7334763

[16] Huan S. Liu M. Liu Z. Gao J., And Yin G. (2023). Association between dietary and serum cholesterol and cognitive function among the U.S. elderly from Nhanes 2011-2014. J Alzheimers Dis 95, 625–640. doi: 10.3233/Jad-230422, PMID: 37574736

[17] Zhang Q. Zhang M. Chen Y. Cao Y., And Dong G. (2022). Nonlinear relationship of non-high-density lipoprotein cholesterol and cognitive function in American elders: a cross-sectional Nhanes study (2011-2014). J Alzheimers Dis 86, 125–134. doi: 10.3233/Jad-215250, PMID: 35001890

[18] Castañer O Pintó X Subirana I Amor AJ Ros E Hernáez Á. Remnant cholesterol, not Ldl cholesterol, is associated with incident cardiovascular disease. J Am Coll Cardiol. (2020) 76:2712–24. doi: 10.1016/J.Jacc.2020.10.008 33272365

[19] Doi T. Langsted A., And Nordestgaard B. G. (2022). Elevated remnant cholesterol reclassifies risk of ischemic heart disease and myocardial infarction. J Am Coll Cardiol 79, 2383–2397. doi: 10.1016/J.Jacc.2022.03.384, PMID: 35710189 PMC8972554

[20] Kontush A. Hdl and reverse remnant-cholesterol transport (Rrt): relevance to cardiovascular disease. Trends Mol Med. (2020) 26:1086–100. doi: 10.1016/J.Molmed.2020.07.005, PMID: 32861590

[21] Qian S You S Sun Y Wu Q Wang X Tang W. Remnant cholesterol and common carotid artery intima-media thickness in patients with ischemic stroke. Circ Cardiovasc Imaging. (2021) 14:E010953. doi: 10.1161/Circimaging.120.010953, PMID: 33832329

[22] Hu X Liu Q Guo X Wang W Yu B Liang B. The role of remnant cholesterol beyond low-density lipoprotein cholesterol in diabetes mellitus. Cardiovasc Diabetol. (2022) 21:117. doi: 10.1186/S12933-022-01554-0 35761281 PMC9238255

[23] Chen M-M Huang X Xu C Song X-H Liu Y-M Yao D. High remnant cholesterol level potentiates the development of hypertension. Front Endocrinol. (2022) 13:830347. doi: 10.3389/Fendo.2022.830347, PMID: 35222285 PMC8863865

[24] Zou Y. Lan J. Zhong Y. Yang S. Zhang H., And Xie G. (2021). Association of remnant cholesterol with nonalcoholic fatty liver disease: a general population-based study. Lipids Health Dis 20,:139. doi: 10.1186/S12944-021-01573-Y, PMID: 34657611 PMC8520640

[25] Paquette M. Bernard S. Paré G., And Baass A. (2022). Dysbetalipoproteinemia: differentiating multifactorial remnant cholesterol disease from genetic Apoe deficiency. J Clin Endocrinol Metab 107, 538–548. doi: 10.1210/Clinem/Dgab648, PMID: 34467996

[26] Ding Z. Luo L. Guo S. Shen Q. Zheng Y., And Zhu S. (2022). Non-linear association between folate/vitamin B12 status and cognitive function in older adults. Nutrients 14,:443. doi: 10.3390/Nu14122443, PMID: 35745173 PMC9227588

[27] Fillenbaum G. G., And Mohs R. (2023). Cerad (consortium to establish a registry for Alzheimer's disease) neuropsychology assessment battery: 35 years and counting. J Alzheimers Dis 93, 1–27. doi: 10.3233/Jad-230026, PMID: 36938738 PMC10175144

[28] Huang J Li R Zhu H Huang D Li W Wang J. Association between serum globulin and cognitive impairment in older American adults. Front Public Health. (2023) 11:1193993. doi: 10.3389/Fpubh.2023.1193993, PMID: 37670828 PMC10476522

[29] Casagrande S. S. Lee C. Stoeckel L. E. Menke A., And Cowie C. C. (2021). Cognitive function among older adults with diabetes and prediabetes, Nhanes 2011-2014. Diabetes Res Clin Pract 178,:108939. doi: 10.1016/J.Diabres.2021.108939, PMID: 34229005 PMC8429258

[30] Weng X Tan Y Fei Q Yao H Fu Y Wu X. Association between mixed exposure of phthalates and cognitive function among the U.S. elderly from Nhanes 2011-2014: three statistical models. Sci Total Environ. (2022) 828:154362. doi: 10.1016/J.Scitotenv.2022.154362, PMID: 35259385

[31] Xie Y-Y Zhao L Gao L-J Xu R-X Gao Y Dou K-F. Association between remnant cholesterol and verbal learning and memory function in the elderly in the us. Lipids Health Dis. (2022) 21:120. doi: 10.1186/S12944-022-01729-4, PMID: 36376895 PMC9664689

[32] Yasamineh S Mehrabani FJ Derafsh E Danihiel Cosimi R Forood AMK Soltani S. Potential use of the cholesterol transfer inhibitor U18666a as a potent research tool for the study of cholesterol mechanisms in neurodegenerative disorders. Mol Neurobiol. (2024) 61:3503–27. doi: 10.1007/S12035-023-03798-7 37995080

[33] Zhang J., And Liu Q. (2015). Cholesterol metabolism and homeostasis in the brain. Protein Cell 6, 254–264. doi: 10.1007/S13238-014-0131-3, PMID: 25682154 PMC4383754

[34] Dunk MM Li J Liu S Casanova R Chen J-C Espeland MA. Associations of dietary cholesterol and fat, blood lipids and risk for dementia in older women vary by apoe genotype. Alzheimers Dement. (2023) 19:5742–54. doi: 10.1002/Alz.13358 37438877 PMC10784407

[35] Ma Y. Liang L. Zheng F. Shi Le Zhong B., And Xie W. (2020). Association between sleep duration and cognitive decline. JAMA Netw Open 3,:E2013573. doi: 10.1001/Jamanetworkopen.2020.13573, PMID: 32955572 PMC7506513

[36] Marchi NA Solelhac G Berger M Haba-Rubio J Gosselin N Vollenweider P. Obstructive sleep Apnoea and 5-year cognitive decline in the elderly. Eur Respir J. (2023) 61:2201621. doi: 10.1183/13993003.01621-2022, PMID: 36796834 PMC10133583

[37] Mason G. M. Lokhandwala S. Riggins T., And Spencer R. M. C. (2021). Sleep And Human Cognitive Development. Sleep Med Rev 57,:101472. doi: 10.1016/J.Smrv.2021.101472, PMID: 33827030 PMC8164994

[38] Banach M. Rizzo M. Nikolic D. Howard G. Howard V., And Mikhailidis D. (2017). Intensive Ldl-cholesterol lowering therapy and neurocognitive function. Pharmacol Ther 170, 181–191. doi: 10.1016/J.Pharmthera.2016.11.001, PMID: 27865998

[39] Hu Y Wang X Lin L Huan J Li Y Zhang L. Association of remnant cholesterol with frailty: findings from observational and Mendelian randomization analyses. Lipids Health Dis. (2023) 22:115. doi: 10.1186/S12944-023-01882-4, PMID: 37537564 PMC10399004

[40] Loera-Valencia R. Goikolea J. Parrado-Fernandez C. Merino-Serrais P., And Maioli S. (2019). Alterations in cholesterol metabolism as a risk factor for developing Alzheimer's disease: potential novel targets for treatment. J Steroid Biochem Mol Biol 190, 104–114. doi: 10.1016/J.Jsbmb.2019.03.003 30878503

[41] Anstey K. J. Ashby-Mitchell K., And Peters R. (2017). Updating the evidence on the association between serum cholesterol and risk of late-life dementia: review and Meta-analysis. J Alzheimers Dis 56, 215–228. doi: 10.3233/Jad-160826, PMID: 27911314 PMC5240556

