# Supplementary material for: Relationship between residual cholesterol and cognitive performance: a study based on NHANES
Source: Front Nutr. 2024 Sep 11;11:1458970. doi: 10.3389/fnut.2024.1458970 (PMC11423777; doi:10.3389/fnut.2024.1458970)
Supplement: Supplementary file 1 [file Table_1.docx]

**Table S1. Patient characteristic.**

| **Characteristic** | **Overall,**  **N = 1331 (100%)^a^** | **Cognitive Function**  **Normal, N = 833 (75%)^a^** | **Cognitive Function**  **Decline, N = 498 (25%)^a^** | **P Value^b^** |
| --- | --- | --- | --- | --- |
| **Age (years)** | 68 (63, 74) | 68 (63, 74) | 69 (65, 76) | 0.082 |
| **Sex** |  |  |  | **0.007** |
| *female* | 685 (56%) | 466 (58%) | 219 (49%) |  |
| *male* | 646 (44%) | 367 (42%) | 279 (51%) |  |
| **Age** |  |  |  | >0.9 |
| *< 70 years* | 709 (58%) | 415 (58%) | 294 (58%) |  |
| *70-80 years* | 401 (28%) | 266 (28%) | 135 (28%) |  |
| *≥80 years* | 221 (14%) | 152 (14%) | 69 (13%) |  |
| **Race, n(%)** |  |  |  | **<0.001** |
| *Non-Hispanic White* | 671 (80%) | 512 (86%) | 159 (61%) |  |
| *Non-Hispanic Black* | 269 (8.3%) | 127 (5.5%) | 142 (17%) |  |
| *Other/multiracial* | 131 (5.0%) | 84 (4.5%) | 47 (6.5%) |  |
| *Mexican American* | 119 (3.5%) | 54 (2.2%) | 65 (7.5%) |  |
| *Other Hispanic* | 141 (3.5%) | 56 (2.0%) | 85 (8.3%) |  |
| **Education level, n(%)** |  |  |  | **<0.001** |
| *Less than high school* | 340 (16%) | 116 (9.5%) | 224 (37%) |  |
| *High school or equivalent* | 314 (22%) | 185 (20%) | 129 (30%) |  |
| *College or above* | 677 (61%) | 532 (70%) | 145 (34%) |  |
| **Marital status, n(%)** |  |  |  | **0.007** |
| *Married* | 786 (65%) | 509 (68%) | 277 (56%) |  |
| *Unmarried* | 545 (35%) | 324 (32%) | 221 (44%) |  |
| **BMI, n(%)** |  |  |  | 0.5 |
| *Underweight (<18.5)* | 17 (1.2%) | 10 (0.9%) | 7 (2.1%) |  |
| *Normal (18.5 to <25)* | 353 (26%) | 219 (26%) | 134 (26%) |  |
| *Overweight (25 to <30)* | 455 (35%) | 285 (36%) | 170 (32%) |  |
| *Obese (30 or greater)* | 506 (38%) | 319 (37%) | 187 (39%) |  |
| **Alcohol, n(%)** |  |  |  | 0.4 |
| *No/Unknown* | 213 (14%) | 124 (13%) | 89 (15%) |  |
| *Yes* | 1,118 (86%) | 709 (87%) | 409 (85%) |  |
| **Smoke status, n(%)** |  |  |  | **0.008** |
| *Current smoker* | 159 (11%) | 78 (8.5%) | 81 (17%) |  |
| *Former smoker* | 507 (40%) | 325 (40%) | 182 (40%) |  |
| *Never smoker* | 665 (49%) | 430 (51%) | 235 (44%) |  |
| **Diabetes, n(%)** |  |  |  | **0.014** |
| *No* | 980 (77%) | 645 (80%) | 335 (68%) |  |
| *Yes* | 351 (23%) | 188 (20%) | 163 (32%) |  |
| **hypertension, n(%)** |  |  |  | 0.2 |
| *No* | 499 (41%) | 321 (43%) | 178 (36%) |  |
| *Yes* | 832 (59%) | 512 (57%) | 320 (64%) |  |
| **Trouble sleeping, n(%)** |  |  |  | 0.7 |
| *Trouble sleeping* | 392 (32%) | 242 (NA%) | 150 (NA%) |  |
| *Non-trouble sleeping* | 939 (68%) | 591 (NA%) | 348 (NA%) |  |
| **Sleep disorder, n(%)** |  |  |  | 0.5 |
| *Sleep disorder* | 165 (13%) | 104 (14%) | 61 (12%) |  |
| *Non-sleep disorder* | 1,166 (87%) | 729 (86%) | 437 (88%) |  |
| **Sleep duration, n(%)** |  |  |  | **0.004** |
| *< 7 hours* | 435 (27%) | 270 (27%) | 165 (28%) |  |
| *7-9 hours* | 772 (65%) | 498 (66%) | 274 (59%) |  |
| *≥9 hours* | 124 (8.4%) | 65 (6.9%) | 59 (13%) |  |
| **CERAD‐WL** | 20 (17, 23) | 22 (19, 24) | 15 (13, 19) | **<0.001** |
| **CERAD‐DR** | 7 (5, 8) | 7 (6, 8) | 4 (3, 5) | **<0.001** |
| **AFT** | 18 (14, 21) | 19 (16, 23) | 14 (11, 16) | **<0.001** |
| **DSST** | 53 (41, 64) | 57 (47, 67) | 36 (27, 45) | **<0.001** |
| **Z-score** | 0.39 (-0.32, 1.00) | 0.70 (0.24, 1.26) | -0.78 (-1.25, -0.34) | **<0.001** |
| **TC, (mg/dL)** | 190 (161, 217) | 193 (165, 220) | 180 (152, 210) | **<0.001** |
| **TG, (mg/dL)** | 105 (75, 152) | 101 (72, 150) | 118 (80, 157) | 0.052 |
| **LDL-C, (mg/dL)** | 109 (86, 132) | 112 (88, 133) | 104 (77, 128) | **0.005** |
| **HDL-C, (mg/dL)** | 54 (45, 66) | 55 (46, 68) | 51 (43, 60) | **<0.001** |
| **RC, (mg/dL)** | 21 (15, 30) | 20 (14, 30) | 24 (16, 31) | **0.048** |
| **TC/RC** | 8.7 (6.0, 12.9) | 9.3 (6.2, 13.4) | 7.7 (5.8, 10.7) | **0.003** |

^a^median (IQR) for continuous; n (%) for categorical, ^b^Wilcoxon rank-sum test for complex survey samples; chi-squared test with Rao & Scott's second-order correction**.** BMI = Body mass index; CERAD-WL = Consortium to Establish a Registry for Alzheimer’s Disease Word Learning; CERAD-DR = CERAD Delayed Recall; AFT = Animal Fluency Test; DSST = Digit Symbol Substitution test; TC = Total cholesterol; TG = Triacylglycerol; LDL-C = Low-density lipoprotein cholesterol; HDL-C = High-density lipoprotein cholesterol; RC = Remnant cholestero
